# Executive Functions in Healthy Older Adults Are Differentially Related to Macro- and Microstructural White Matter Characteristics of the Cerebral Lobes

**DOI:** 10.3389/fnagi.2017.00373

**Published:** 2017-11-30

**Authors:** Sarah Hirsiger, Vincent Koppelmans, Susan Mérillat, Cornelia Erdin, Atul Narkhede, Adam M. Brickman, Lutz Jäncke

**Affiliations:** ^1^International Normal Aging and Plasticity Imaging Center, University of Zurich, Zurich, Switzerland; ^2^University Research Priority Program Dynamics of Healthy Aging, University of Zurich, Zurich, Switzerland; ^3^Department of Psychiatry, University of Utah, Salt Lake City, UT, United States; ^4^School of Kinesiology, University of Michigan, Ann Arbor, MI, United States; ^5^Taub Institute for Research on Alzheimer's Disease and the Aging Brain, Department of Neurology, College of Physicians and Surgeons, Columbia University, New York, NY, United States; ^6^Division of Neuropsychology, University of Zurich, Zurich, Switzerland; ^7^Department of Special Education, King Abdulaziz University, Jeddah, Saudi Arabia

**Keywords:** diffusion tensor imaging, white matter hyperintensities, aging, executive functions, processing speed, multimodal imaging

## Abstract

Aging is associated with microstructural white matter (WM) changes. WM microstructural characteristics, measured with diffusion tensor imaging (DTI), are different in normal appearing white matter (NAWM) and WM hyperintensities (WMH). It is largely unknown how the microstructural properties of WMH are associated with cognition and if there are regional effects for specific cognitive domains. We therefore examined within 200 healthy older participants (a) differences in microstructural characteristics of NAWM and WMH per cerebral lobe; and (b) the association of macrostructural (WMH volume) and microstructural characteristics (within NAWM and WMH separately) of each lobe with measures of executive function and processing speed. Multi-modal imaging (i.e., T1, DTI, and FLAIR) was used to assess WM properties. The Stroop and the Trail Making Test were used to measure inhibition, task-switching (both components of executive function), and processing speed. We observed that age was associated with deterioration of white matter microstructure of the NAWM, most notably in the frontal lobe. Older participants had larger WMH volumes and lowest fractional anisotropy values within WMH were found in the frontal lobe. Task-switching was associated with cerebral NAWM volume and NAWM volume of all lobes. Processing speed was associated with total NAWM volume, and microstructural properties of parietal NAWM, the parietal WMH, and the temporal NAWM. Task-switching was related to microstructural properties of WMH of the frontal lobe and WMH volume of the parietal lobe. Our results confirm that executive functioning and processing speed are uniquely associated with macro- and microstructural properties of NAWM and WMH. We further demonstrate for the first time that these relationships differ by lobar region. This warrants the consideration of these distinct WM indices when investigating cognitive function.

## Introduction

Aging is associated with macro- and microstructural white matter (WM) changes (Gunning-Dixon et al., 2009; Bai et al., 2011). These changes are related or even mediate age-related cognitive changes, particularly executive functioning and processing speed (Raz and Rodrigue, 2006; Perry et al., 2009; Fjell and Walhovd, 2010; Gold et al., 2010). Most studies that investigate the relationship between WM and cognition examine either microstructural WM characteristics or macrostructural WM properties (O'sullivan et al., 2001; Prins et al., 2005; Kennedy and Raz, 2009). WM microstructure can be assessed by measuring the diffusion of water molecules within WM. Using diffusion tensor imaging (DTI) orientation and quantity of water motion within tissue can be calculated (Mori and Zhang, 2006). In aging research, the most conclusive results are found for fractional anisotropy (FA) and mean diffusivity (MD). In general, aging is associated with FA decreases an MD increases. These aging effects are most pronounced in anterior brain regions (Sullivan and Pfefferbaum, 2006; Madden et al., 2009). Several studies that investigated WM volume did not regard macrostructural WM abnormalities, typically detected with T2-weighted MRI, and described as white matter hyperintensities (WMH). Some studies, however, looked specifically at WMH (number or volume). WMH are linked to myelin and axonal degeneration, gliosis, and small vessel disease (Gouw et al., 2011). There is evidence that WMH volume is associated with the magnitude of WM microstructural degeneration within NAWM, suggesting that macro and microstructural WM changes are different states of the same neurodegenerative process (Maillard et al., 2013; Pelletier et al., 2015). Teasing apart the association between microstructure of WMH and cognition and microstructure of NAWM and cognition could improve our understanding of brain structural-behavioral relationships. Moreover, it could provide additional insight in the unique contribution of microstructural alterations next to the well-described macrostructural influences (e.g., WMH and NAWM volume) on cognition. The few studies that have reported diffusion metrics separately for NAWM and WMH not only found lower FA values and higher diffusion values within WMH (Vernooij et al., 2009; Maniega et al., 2015), but also reported that, on a whole brain level, associations between cognitive abilities and microstructural integrity are different within NAWM and WMH (Vernooij et al., 2009; Schmidt et al., 2010; Jokinen et al., 2013).

Because of the association between global deterioration of WM microstructure and processing speed (Salthouse, 1996; Eckert et al., 2010), a whole brain analysis approach is suitable for this cognitive function. However, considering that executive functioning is primarily mediated by the frontal lobes, conducting regional analyses can provide additional insight into cognitive functioning and its relation to WM alterations with aging. Furthermore, the prefrontal-executive theory of cognitive aging postulates that disproportional age-related frontal lobe deterioration mediates executive function decline (West, 1996), but the relationship between WM characteristics and executive function is not yet fully understood. This is likely due to lack of standardization of the definition and conceptualization of executive function and its subcomponents (e.g., Miyake et al., 2000). Although performance on different executive function subcomponent tests has been associated with different WM structures (Kennedy and Raz, 2009) previous studies have often combined different executive function components into a single compound score (Grieve et al., 2007; He et al., 2012), which further hampers interpretation.

In the current study we relied on the concepts proposed by Miyake et al. (2000) who demonstrated that three basic psychological functions best describe the core elements of executive function: inhibition, shifting, and updating. We examined the association between executive functioning and microstructural WM properties within NAWM and WMH separately. To investigate regional effects for specific cognitive domains (especially executive function) we decided to stratify our analysis by cerebral lobes. Macrostructural and microstructural indices were obtained from T2-weighted fluid-attenuated inversion recovery (FLAIR) and DTI sequences from 200 highly educated healthy older participants. First, we investigated the effects of age on white matter microstructure, macrostructure, and cognition. In a second step, we determined (a) if FA and MD values in NAWM and WMH are different across cerebral lobes; (b) if there are differences in the volume of WMH between cerebral lobes; and (c) if there is a difference in the values of FA and MD between cerebral lobes (this analysis was stratified by NAWM and WMH). Lastly, we examined the association of microstructural and macrostructural properties of NAWM and WMH within the entire cerebrum and within each cerebral lobe with measures of executive function (i.e., inhibition and task-switching) and processing speed. Processing speed was chosen as a reference measure because its aging-related decline has been associated with rather global alterations of WM microstructure (Salthouse, 1996; Eckert et al., 2010). This makes processing speed well suited to contrast the more regionally specific measures of executive functioning. Because of gender differences on brain structure and cognitive function (Maylor et al., 2007; Sachdev et al., 2009; Cox et al., 2016; Rathee et al., 2016) in older participants, we additionally explore the effect of (a) gender and gender by age interactions with WM structure; and (b) between gender and gender by WM structure interactions with cognitive function.

By investigating the association between macrostructural and microstructural WM properties of WMH and NAWM per cerebral lobe with core elements of executive function we aim to obtain a deeper understanding of cognitive functioning in healthy older adults. We hypothesized that within anterior brain regions FA of WMH would be lower and MD of WMH would be higher as those values within WMH of posterior brain regions, similar to the pattern that has been observed in NAWM (Pfefferbaum et al., 2005). Furthermore, we hypothesized that cognitive performance would be positively associated with FA and negatively associated with MD within NAWM and WMH. More specifically, we expected that executive function performance would be associated with WM characteristics in the frontal lobe (but not other lobes) and that processing speed would be associated with global and lobar WM properties.

## Materials and methods

### Participants

This cross-sectional study includes the baseline neuroimaging (DTI, FLAIR) and cognitive performance data from 200 subjects from the Longitudinal Healthy Aging Brain (LHAB) database project. This longitudinal study is being conducted at the International Normal Aging and Plasticity Imaging Center (INAPIC) and University Priority Program “Dynamics of Healthy Aging” at the University of Zurich (Zöllig et al., 2011). The mean age of the sample is 70.54 ± 4.88 years (106 women, 94 men). Subjects had to meet the following inclusion criteria: age >64, Mini Mental State Examination (MMSE, Folstein et al., 1975) score >26, German native speaker, right-handed, no self-reported history of neurological/psychiatric disease, and no contraindications for MRI. The local ethics committee (Kantonale Ethikkommission Zurich) approved the study in accordance with guidelines from the Helsinki declaration and all participants gave written informed consent.

### Neuropsychological tests

For a deeper understanding of executive function we analyzed two subcomponents i.e., inhibition and task-switching.

Inhibition performance was assessed using a computerized version of the Stroop task (Vienna Test System, Schuhfried, 2009, Version 23, S8). One hundred word stimuli (25 congruent, e.g., “red” displayed in red font, and 75 incongruent words, e.g., “red” displayed in blue font) were randomly presented on a screen. The participants were instructed to indicate the color in which the letters were displayed as fast as possible by pressing the corresponding color button from the four color options (red, green, blue and yellow). Median reaction times for the congruent stimuli (RT_C_) and for the incongruent stimuli (RT_I_) were compared. A difference score for inhibition performance (IP_Diff_) was computed (RT_I_-RT_C_).

Task-switching performance was measured with the Trail Making Test Parts A and B (TMT, Reitan and Wolfson, 1985). In part A the goal is to connect ascending numbers on a sheet of paper in the correct order as fast as possible. In part B in contrast to part A, not only numbers but also letters have to be connected alternately (e.g., 1, A, 2, B …). For task-switching a difference scores was calculated (TMT_Diff_ = TMT(B)–TMT(A)).

Processing speed (PS) ability was measured by the reaction time RT_C_ of the Stroop task.

### MRI acquisition

MRI was performed on a Philips Ingenia 3T scanner. For structural imaging, two T1-weighted (T1) sagittal scans were collected with a 3D Turbo-Field-Echo (TFE) sequence (TR = 8.18ms, TE = 3.799 ms, field of view (FoV) = 240 × 240 mm, acquisition matrix = 240 × 240 mm, slice thickness = 1 mm, 160 slices, 1 mm^3^ isotropic voxel, flip angle = 8°, number of signal average (NSA) = 1, duration~7:30 min.). The DTI scan consisted of a single-shot echo-planar (EPI) sequence (TR = 23.983 s, TE = 55 ms, FoV = 224 × 224 mm, acquisition matrix = 112 × 112 mm, slice thickness = 2 mm, 75 contiguous slices, 2 mm^3^ isotropic voxel, flip angle = 90°, Echo Train Length (ETL) = 59, NSA = 1). One non-weighted image (*b*-value = 0 s/mm^2^), and 32 diffusion-weighted directions with a maximum *b*-value of 1,000 s/mm^2^ were obtained. The diffusion-weighted directions were equally distributed in space. Acquisition time was ~15 min. Additionally, a fluid-attenuated inversion recovery (FLAIR) sequence was collected (TR = 11 s, TE = 125 ms, TI = 2.800 s, FoV = 240 × 180 mm, acquisition matrix = 368 × 186, slice thickness = 4 mm, 32 slices, voxel size = 0.65 × 0.97 × 4 mm^3^, NSA = 1, scan time ~2 min).

### Image preprocessing

#### Diffusion tensor imaging

DTI images were processed with FMRIB's diffusion toolbox (FDT) (FMRIB Software Library (FSL), (http://fsl.fmrib.ox.ac.uk/fsl/fslwiki) (Smith et al., 2004) by executing the following steps: First, data were corrected for head motion artifacts and eddy currents. Second, a binary brain mask was created based on the first recorded non-diffusion weighted (*b* = 0) image with FSL's brain extraction tool (BET) applying a fractional intensity threshold of 0.3. Diffusion tensors were fitted locally at each voxel using dtifit, which yielded Fractional Anisotropy (FA), and Mean Diffusivity (MD). T1 images were averaged using FSL's *AnatomicalAverage* script. Prior to averaging, all T1 images were neck-stripped (under the cerebellum) to enhance averaging accuracy. Magnetic field inhomogeneity artifacts were corrected within the brain area of the averaged T1 and FLAIR images by applying N4ITK (Tustison et al., 2010) within a binary brain mask obtained using FSL's BET with a fractional intensity threshold of 0.3 and robust brain center estimation.

#### White matter hyperintensities (WMH)

White matter hyperintensity volume was quantified according to an adapted version of the approach developed in house (Brickman et al., 2009, 2011). Briefly, Gaussian curves were fit to the distribution of each subject's FLAIR voxel intensity values. White matter hyperintensities were defined as those voxels with an intensity higher than 1.7 standard deviations above the mean. After applying the threshold, all images were further inspected visually and errors were corrected manually by two of the authors (CE and AN).

#### Total white matter

To assess the volume of cerebral white matter we first constructed a cerebrum mask by subtracting FreeSurfer's cerebellum, brainstem and 4th ventricle maps from FreeSurfer's binarized white matter parcellation map (wmparc). The resulting image was dilated and voids that did not touch the edge of the FoV were filled. Finally, FreeSurfer's white matter segmentation map (wm.seg) was masked with the cerebrum mask and an intracranial volume (ICV) mask that was created by combining FSL's BET and tissue class segmentations to obtain a binary map of the cerebral white matter from which the total cerebral WM volume was than derived by multiplying the number of voxels in the mask by the voxel size.

#### DTI parameters within the WMH and the NAWM

**Figure 1:**
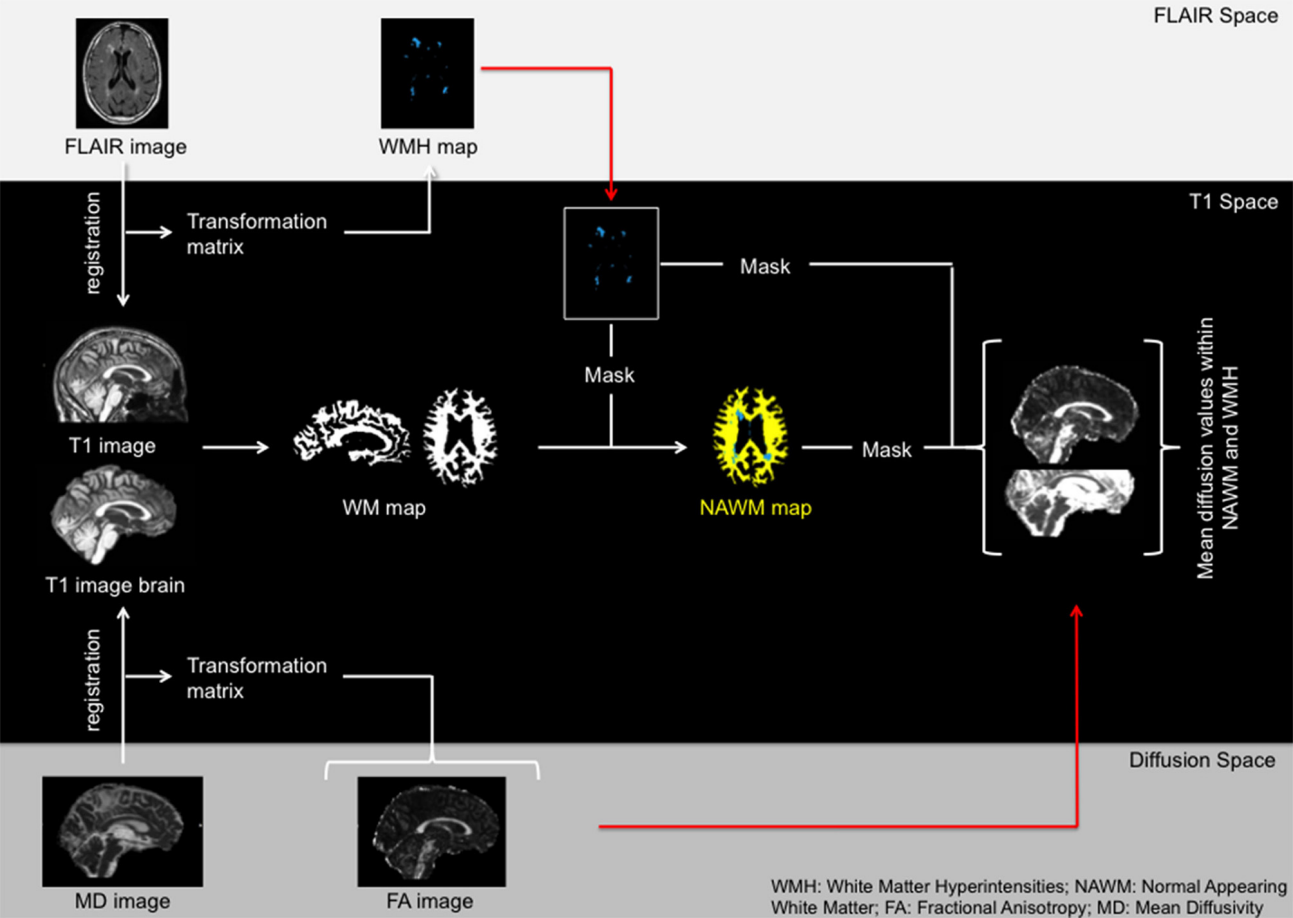
Overview of the registration process.

To compare microstructural alterations within WMH and NAWM we divided the voxels within the total white matter mask to either one of the two classes. First, the FLAIR images and T1-weighed images were co-registered with FMRIB's Linear Image Registration Tool (FLIRT) with mutual information as cost function. The transformation matrix was then applied to the WMH maps with nearest neighbor interpolation. Second, skull striped MD and T1 images were co-registered using FLIRT with mutual information as cost function. The acquired transformation matrix was then applied to all other DTI images. Both registrations were visually checked. The MD map was chosen as the reference image since it was previously shown to yield optimal registration results (Vernooij et al., 2009). The average values of the DTI metrics within the WMH or the NAWM were calculated by averaging the values of all voxels in their respective masks. Figure 1 displays an overview of the preprocessing and masking steps.

#### Lobar volumes and DTI parameters within NAWM and WMH

We obtained macrostructural and microstructural WM characteristics of WMH and NAWM, as well as WMH volumes (section White Matter Hyperintensities (WMH)) of each cerebral lobe separately. For this purpose binary masks for the cerebral lobes (i.e., frontal, parietal, temporal, and occipital) were obtained for each individual using NeuroAnalytica (formerly Brain Research: Analysis of Images Networks and Systems; BRAINS) (Pierson et al., 2011; Li et al., 2013). NeuroAnalytica uses atlas registration to divide the brain into lobes and the cerebellum. This multi-step approach first segments the T1 image into gray matter (GM) and WM discriminant images. A WM template is then registered to the subject's WM discriminant image in five steps from rigid to nonlinear. The resulting deformation field is used as a starting point for nonlinear registration of a GM template to the subject's GM discriminant image. The deformation field obtained in this last step is applied to a lobar atlas that is part of NeuroAnalytica/BRAINS. These lobar masks that were derived from the subject specific atlas were then used to mask out the NAWM and WMH volume as well as the DTI metrics within NAWM and WMH for each lobe.

### Statistical analysis

Data analysis was conducted using IBM SPSS Statistics for Mac OSX, Version 22. For dependent and independent variables data three standard deviations (SD) above or below the mean were considered outliers and removed from the analysis to prevent that these cases were driving the associations. White matter hyperintensity volumes were natural log-transformed because of skewness. In addition, all variables of interest (i.e., micro- and macrostructural WM measures, executive function and processing speed scores) were standardized to z-scores. Behavioral data was reversed (multiplied by −1) if necessary so that higher numbers always represent better performance. Because former studies found gender differences in diffusion values as well as WM and WMH volume in aging samples we included gender as a covariate of interest in our models.

Relationships between age and cognition measures were evaluated using Pearson correlations. To analyze if microstructural integrity within and outside WMHs differ we applied two-tailed paired sample *t*-tests.

We used hierarchical regression analysis to analyze the association between age and WM macro- and microstructural characteristics per cerebral lobe. Predictor variables of interest included in the primary model were age and gender. In the extended model, we added a gender^*^age interaction term to analyze if the slope of these WM characteristics differentially changes over time for males and females.

Linear mixed models (LMM) with random intercept for subject were used to evaluate lobar differences in (1) diffusion values within WMH/NAWM and (2) WMH burden (WMH volumes depicted as ratio of ICV).

Hierarchical regression models were used to investigate the association between WM characteristics and cognitive performance (dependent variable). Predictor variables of interest included in the primary model were WM characteristics and gender. If gender had a significant effect and/or we found that WM characteristics explained significant variance we extended the model. In our extended model, we added a gender^*^WM characteristic interaction term to analyze if the association between WM characteristics and cognition was different males and females. Only if the interaction term was significant and if there was a significant model improvement from the initial model to the extended model, measured by a significant R^2^ change, do we report on the interaction effects.

### Covariates

All linear and regression models were controlled for education and diastolic blood pressure, because they are known confounders in the relationships between aging, cognition and and/or WM integrity (Alley et al., 2007; Marcus et al., 2011; Beauchet et al., 2013; Noble et al., 2013). Years of education was determined by self-report. Blood pressure was measured three times on the same day on the left arm, while subjects were seated and in upright position with the arm at rest using a blood pressure cuff (Model M6, HEM-7211-E; Omron Corporation, Kyoto, Japan). The average diastolic blood pressure was used for analysis. Because women have significant smaller brain volumes than men (Ruigrok et al., 2014), and because brain size has been shown to have strong effects on volume measures (Jancke et al., 2015), models incorporating NAWM or WMH volume were additionally adjusted for ICV, which obtained by FreeSurfer (estimated total ICV). Models investigating the association between diffusion values and cognition were also adjusted for NAWM and WMH volume to evaluate if the associations are independent from WMH volume as well as atrophy.

The level of significance for all tests was set to *p* < 0.05. Age associations, LMM as well as the *t*-tests were adjusted for multiple comparisons using the procedure suggested by Holm (1979). Eta-squared (η^2^) was calculated as measure of effect size for the hierarchical regression models by dividing the Type III Sum of Squares of the factor of interest by the Total Sum of Squares (“Corrected Total” in SPSS). In addition, phi (ϕ) was calculated as effect size measure for our LMM by taking the square root from the ratio of the obtained chi-square value and the number of observations.

## Results

Sample characteristics are presented in Table 1. All subjects had WMH in the frontal lobe whereas nine, four, and one subjects did not have measurable WMH in the temporal, parietal and occipital lobe, respectively. Comparing diffusion values in NAWM to diffusion values in WMH for each lobe we found that FA values within NAWM were significantly higher than FA values within WMH in the frontal and parietal lobe [Cerebrum: *t*_(196)_ = 26.11, *p* < 0.001; Frontal: *t*_(195)_ = 47.60, *p* < 0.001; Parietal: *t*_(193)_ = 5.96, *p* < 0.001; Temporal: *t*_(186)_ = −1.47, *p* = 0.143; Occipital: *t*_(194)_ = −21.41, *p* < 0.001] and MD values were lower across the cerebrum and within single lobes [Cerebrum: *t*_(196)_ = 26.11; Frontal: *t*_(195)_ = −44.56; Parietal: *t*_(191)_ = −38.95; Temporal: *t*_(186)_ = −38.31; Occipital: *t*_(194)_ = −33.18 (all *p* < 0.001)].

**Table 1:** Characteristics of the study population.

**Variable**	**N**	**M (SD)**
Age	200	70.54 (4.88)
Men (n (%))	200	94 (46%)
Years of education completed	200	14.63 (3.37)
Blood Pressure diastolic (mmHg)	200	81.35 (10.02)
MMSE	200	28.87 (0.99)
*Processing speed:*		
RT_C_ (sec)	197	0.977 (0.165)
*Inhibition:*		
IP_Diff_	197	0.108 (0.098)
*Task-switching:*		
TMT_Diff_ (sec)	195	51.744 (23.840)

*IP_Diff_, inhibition performance difference score; M, mean; MMSE, Mini Mental State Examination; mmHg, millimeter of mercury; RT_C_, median reaction time for congruent stimuli; SD, standard deviation; sec, seconds; TMT, trail making test*.

### Age effects on white matter microstructure, macrostructure, and cognition

Age was negatively associated with whole brain and all lobar volume measures for NAWM, and positively associated with whole brain, frontal, and parietal WMH volumes (Table 2). Females had less NAWM volume in the parietal and temporal lobe than males. Effect sizes for ICV in these models were η^2^ = 0.174 and η^2^ = 0.160 for parietal and temporal NAWM volume, respectively. By removing ICV from the model to evaluate the effect of ICV in comparison to gender the effect sizes for gender increased from η^2^ = 0.029 to η^2^ = 0.257 and from η^2^ = 0.028 to η^2^ = 0.237 for parietal and temporal NAWM volume, respectively. These results survived multiple comparison correction.

**Table 2:** Association between age, gender and white matter macrostructural measures.

		**N**	**Mean (SD)**	**Age**			**Gender**_**(female)**_		
				**B**	**SE**	**η^2^**	**B**	**SE**	**η^2^**
**CEREBRUM**									
Vol.	NAWM	200	462.318 (54.911)	−0.069^**^^¶^	0.010	0.109	−0.326^**^	0.121	0.016
	WMH^a^	196	2.399 (1.181; 4.456)	0.051^**^^¶^	0.014	0.059	0.388^*^	0.175	0.023
**FRONTAL**									
Vol.	NAWM	199	193.720 (24.024)	−0.074^**^^¶^	0.010	0.127	−0.162	0.128	0.004
	WMH^a^	196	1.397 (0.670; 2.410)	0.045^**^^¶^	0.014	0.046	0.468^**^	0.177	0.033
**PARIETAL**									
Vol.	NAWM	200	105.413 (13.149)	−0.049^**^^¶^	0.010	0.056	−0.439^**^^¶^	0.129	0.029
	WMH^a^	190	0.315 (0.123; 0.856)	0.053^**^^¶^	0.015	0.064	0.336	0.177	0.017
**TEMPORAL**									
Vol.	NAWM	200	78.784 (9.632)	−0.074^**^^¶^	0.010	0.126	−0.428^**^^¶^	0.126	0.028
	WMH^a^	188	0.120 (0.043; 0.350)	0.024	0.015	0.012	0.302	0.180	0.014
**OCCIPITAL**									
Vol.	NAWM	200	60.378 (8.383)	−0.055^**^^¶^	0.011	0.070	−0.426^**^	0.138	0.028
	WMH^a^	194	0.328 (0.158; 0.663)	0.022	0.015	0.011	−0.197	0.180	0.006

*NAWM, normal appearing white matter; SD, standard deviation; SE, standard error; WMH, white matter hyperintensity; Vol., volume. Volumes for WMH and NAWM are indicated in ml*.

* p < 0.05;

** p < 0.01;

¶ *corrected for multiple comparisons*.

*All models were adjusted for education, diastolic blood pressure, and intracranial volume*.

a *natural log transformed*.

After multiple comparison correction, age remained significantly associated with FA decreases in the NAWM of the frontal lobe and MD increases within NAWM in all lobes as well as on a whole brain level. For diffusion metric within WMH we observed corrected significant FA increases with age within the frontal, and temporal lobe and an increase in MD within the occipital lobe. Effects of gender on WM microstructural characteristics did not survive multiple comparisons correction. The largest effect sizes for the association between age and WMH characteristics (volume, FA as well as MD) were found in the frontal lobe (Table 3).

**Table 3:** Association between age, gender and white matter microstructural measures.

		**N**	**Mean (SD)**	**Age**			**Gender**_**(female)**_			**Age** × **Gender**		
				**B**	**SE**	**η^2^**	**B**	**SE**	**η^2^**	**B**	**SE**	**η^2^**
**CEREBRUM**												
FA	NAWM	197	3.3 × 10^−1^(1.5*x*10^−2^)	−0.041^**^	0.014	0.040	−0.201	0.147	0.009			
	WMH	200	2.7 × 10^−1^(3.3 × 10^−2^)	0.031^*^	0.014	0.022	−0.261	0.146	0.015			
MD	NAWM	196	8.1 × 10^−4^(2.8 × 10^−5^)	0.100^**^^¶^	0.013	0.235	−0.073	0.130	0.001			
	WMH	197	1.3 × 10^−3^(1.3 × 10^−4^)	−0.002	0.015	< 0.001	0.130	0.149	0.004			
**FRONTAL**												
FA	NAWM	197	3.3 × 10^−1^(1.5 × 10^−2^)	−0.047^**^^¶^	0.014	0.052	−0.188	0.146	0.008			
	WMH	198	2.2 × 10^−1^(3.0 × 10^−2^)	0.049^**^^¶^	0.014	0.057	−0.156	0.143	0.006			
MD	NAWM	196	8.3 × 10^−4^(3.2 × 10^−5^)	0.102^**^^¶^	0.013	0.244	0.008	0.129	< 0.001			
	WMH	199	1.4 × 10^−3^(1.8 × 10^−4^)	−0.017	0.015	0.007	−0.106	0.148	0.003			
**PARIETAL**												
FA	NAWM	198	3.5 × 10^−1^(1.9 × 10^−2^)	−0.033^*^	0.014	0.026	−0.284	0.147	0.018			
	WMH	196	3.3 × 10^−1^(5.3 × 10^−2^)	0.003	0.021	< 0.001	−4.198^*^	2.064	0.021	0.059^*^	0.029	0.020
MD	NAWM	196	7.9 × 10^−4^(2.2 × 10^−5^)	0.086^**^^¶^	0.013	0.175	0.085	0.136	0.002			
	WMH	195	1.3 × 10^−3^(1.7 × 10^−4^)	−0.017	0.015	0.006	0.163	0.149	0.006			
**TEMPORAL**												
FA	NAWM	198	3.3 × 10^−1^(1.8 × 10^−2^)	−0.040^**^	0.014	0.038	0.072	0.147	0.001			
	WMH	189	3.3 × 10^−1^(6.7 × 10^−2^)	0.059^**^^¶^	0.015	0.080	−0.097	0.146	0.002			
MD	NAWM	196	8.2 × 10^−4^(2.9 × 10^−5^)	0.102^**^^¶^	0.020	0.109	3.504	1.899	0.014	−0.057^*^	0.027	0.018
	WMH	189	1.3 × 10^−3^(1.7 × 10^−4^)	−0.022	0.015	0.011	0.104	0.151	0.003			
**OCCIPITAL**												
FA	NAWM	198	2.6 × 10^−1^(1.6 × 10^−2^)	−0.032^*^	0.015	0.024	−0.172	0.147	0.007			
	WMH	197	3.3 × 10^−1^(3.7 × 10^−2^)	0.001	0.015	< 0.001	0.130	0.150	0.004			
MD	NAWM	197	7.9 × 10^−4^(3.1 × 10^−4^)	0.113^**^^¶^	0.019	0.146	3.932^*^	1.848	0.018	−0.058^*^	0.026	0.020
	WMH	197	1.1 × 10^−3^(1.2 × 10^−4^)	0.057^**^^¶^	0.014	0.076	−0.024	0.145	< 0.001			

*FA, fractional anisotropy; MD, mean diffusivity; NAWM, normal appearing white matter; SD, standard deviation; SE, standard error; WMH, white matter hyperintensity*.

* p < 0.05;

** p < 0.01;

¶ *corrected for multiple comparisons*.

*All models were adjusted for education, and diastolic blood pressure*.

Lobar differences in regional macro- and microstructural white matter are shown in Table 4. Results showed that the NAWM FA value within the occipital lobe was lower than FA values within the frontal, parietal, and temporal lobe, so as the WMH MD value within the occipital lobe compared to frontal, parietal, and temporal lobe (ϕ range: 0.121–0.214). In contrasts, NAWM MD values between different lobes as well as WMH FA values within distinct lobes differed less (ϕ range: 0.080–0.123) and findings were inconsistent. For WMH largest volume was found in the frontal lobe, however, this finding did not survive the correction for multiple comparisons.

**Table 4:** Differences in white matter characteristics between cerebral lobes.

		**Frontal vs. Parietal**	**Frontal vs. Temporal**	**Frontal vs. Occipital**	**Parietal vs. Temporal**	**Parietal vs. Occipital**	**Temporal vs. Occipital**
**NAWM**							
FA^b^	Contrast	−0.014	0.013	0.085^**^^¶^	0.026	0.100^**^^¶^	0.072^**^^¶^
	SE	0.016	0.016	0.016	0.016	0.016	0.016
	χ^2^	0.69	0.61	27.03	2.60	36.31	19.40
	ϕ	0.030	0.028	0.185	0.057	0.214	0.157
	p	0.406	0.434	<0.001	0.107	<0.001	<0.001
MD^b^	Contrast	2.4 × 10^−5^	−3.3 × 10^−5^	−2.1 × 10^−5^	−5.7 × 10^−5^^*^	−4.5 × 10^−5^	1.2 × 10^−5^
	SE	2.9 × 10^−5^	2.9 × 10^−5^	2.9 × 10^−5^	2.9 × 10^−5^	2.9 × 10^−5^	2.9 × 10^−5^
	χ^2^	0.72	1.28	0.53	3.90	2.49	0.16
	ϕ	0.030	0.040	0.026	0.070	0.056	0.014
	p	0.396	0.258	0.465	0.048	0.114	0.685
**WMH**							
Vol.^a^^,^^b^	Contrast	3.369^*^	1.639	−0.444	−1.730	−3.812^*^	−2.082
	SE	1.621	1.636	1.615	1.645	1.625	1.632
	χ^2^	4.32	1.00	0.08	1.11	5.50	1.63
	ϕ	0.075	0.036	0.010	0.038	0.085	0.046
	p	0.038	0.317	0.784	0.293	0.019	0.202
FA^b^	Contrast	−0.065	0.113	−0.172^*^	0.0178^*^	−0.108	−0.285^**^^¶^
	SE	0.077	0.079	0.077	0.079	0.077	0.079
	χ^2^	0.70	2.07	4.98	5.09	1.94	13.16
	ϕ	0.030	0.051	0.080	0.081	0.050	0.130
	p	0.403	0.150	0.026	0.024	0.164	<0.001
MD^b^	Contrast	1.4 × 10^−4^	3.2 × 10	1.2 × 10^−3^^**^^¶^	1.9 × 10^−4^	1.1 × 10^−3^^**^^¶^	8.8 × 10^−4^^**^^¶^
	SE	2.5 × 10^−4^	2.6 × 10^−4^	2.6 × 10^−4^	2.6 × 10^−4^	2.6 × 10^−4^	2.6 × 10^−4^
	χ^2^	0.28	1.55	22.06	0.52	17.28	11.45
	ϕ	0.019	0.045	0.168	0.026	0.149	0.121
	p	0.595	0.213	<0.001	0.471	<0.001	<0.001

*FA, fractional anisotropy; MD, mean diffusivity; NAWM, normal appearing white matter; SE, standard error; Vol., volume; WMH, white matter hyperintensity*.

* p < 0.05;

** p < 0.01;

¶ *corrected for multiple comparisons*.

a *Natural log transformed and divided by ICV*.

b *Adjusted for age, gender, education, and blood pressure*.

Age was associated with executive function scores and processing speed measures [IP_Diff_: *r*_(195)_ = −0.24; TMT_Diff_: *r*_(193)_ = −0.22; RT_C_: *r*_(195)_ = −0.31; (*p* < 0.001–0.003)].

### Associations between cognition and white matter characteristics

Associations between WM characteristics and task-switching are summarized in Table 5. No associations were found between WM characteristics or volume with inhibition performance. Better task-switching performance was associated with larger NAWM volumes across the brain but not with WM microstructural characteristics within NAWM. In contrast, larger WMH volume within the parietal lobe was related to worse task-switching performance, as were diffusion metrics within WMH in the frontal lobe. These results did not survive multiple comparisons correction. Gender did not moderate the association between white matter structure and task-switching performance.

**Table 5:** Effects between WM characteristics and task-switching performance.

		**Cerebrum**	**Frontal**	**Parietal**	**Temporal**	**Occipital**
NAWM Volume^b^	B	**0.300**	**0.248**	**0.263**	**0.260**	**0.198**
	SE	0.102	0.098	0.097	0.099	0.092
	η^2^	0.040	0.030	0.035	0.032	0.022
	p	0.004	0.012	0.007	0.010	0.033
WMH Volume^a^^,^^b^	B	−0.097	−0.077	−**0.154**	−0.005	−0.052
	SE	0.075	0.074	0.076	0.074	0.072
	η^2^	0.008	0.005	0.021	<0.001	0.003
	p	0.197	0.301	0.043	0.943	0.471
**NAWM**						
FA^c^	B	−0.081	−0.110	−0.055	0.048	*−0.132*
	SE	0.074	0.076	0.075	0.072	0.071
	η^2^	0.006	0.010	0.003	0.002	0.016
	p	0.277	0.151	0.464	0.507	0.065
MD^c^	B	0.025	0.004	0.033	0.001	0.019
	SE	0.086	0.088	0.081	0.081	0.080
	η^2^	<0.001	<0.001	<0.001	<0.001	<0.001
	p	0.776	0.959	0.686	0.987	0.810
**WMH**						
FA^c^	B	−0.064	−0.013	−0.031	0.023	−0.050
	SE	0.070	0.073	0.072	0.079	0.070
	η^2^	0.004	<0.001	<0.001	<0.001	0.002
	p	0.362	0.857	0.669	0.771	0.477
MD^c^	B	*−0.141*	−**0.179**	−0.061	−0.047	−0.101
	SE	0.076	0.082	0.078	0.073	0.077
	η^2^	0.016	0.022	0.003	0.002	0.008
	p	0.067	0.030	0.435	0.519	0.193

*FA, fractional anisotropy; MD, mean diffusivity; NAWM, normal appearing white matter; SE, standard error; WMH, white matter hyperintensity*.

*Significant results are indicated in bold, trends are indicated in italic*.

a *Natural log transformed*.

b *Adjusted for age, gender, education, blood pressure, and intracranial volume*.

c *Adjusted for age, gender, education, blood pressure, intracranial volume, NAWM Volume, and WMH Volume*.

Associations between WM characteristics and processing speed performance are summarized in Table 6. On the whole brain level, processing speed was associated with NAWM volume as well as MD within NAWM and WMH. Faster processing speed was furthermore associated with larger NAWM volumes and lower MD within NAWM in the parietal and temporal lobe. FA values within parietal WMH were also associated with better processing speed performance. These results did not survive multiple comparisons correction. No gender effects were found in the association between processing speed and WM characteristics.

**Table 6:** Effects between WM characteristics and processing speed performance.

		**Cerebrum**	**Frontal**	**Parietal**	**Temporal**	**Occipital**
NAWM Volume^b^	B	**0.213**	0.147	**0.203**	**0.219**	0.114
	SE	0.103	0.099	0.097	0.100	0.092
	η^2^	0.020	0.010	0.020	0.022	0.007
	p	0.040	0.139	0.038	0.030	0.215
WMH Volume^a^^,^^b^	B	−0.096	−0.103	−0.058	−0.035	0.001
	SE	0.073	0.072	0.074	0.076	0.071
	η^2^	0.008	0.010	0.003	0.001	<0.001
	p	0.190	0.155	0.438	0.645	0.992
**NAWM**						
FA^c^	B	0.068	0.075	0.002	0.044	0.015
	SE	0.074	0.075	0.075	0.071	0.071
	η^2^	0.004	0.005	<0.001	0.002	<0.001
	p	0.359	0.321	0.980	0.541	0.831
MD^c^	B	−**0.169**	*−0.159*	−**0.175**	−**0.182**	−0.098
	SE	0.084	0.086	0.079	0.079	0.079
	η^2^	0.020	0.017	0.024	0.026	0.008
	p	0.047	0.065	0.028	0.022	0.215
**WMH**						
FA^c^	B	0.099	0.052	**0.153**	−0.011	0.036
	SE	0.069	0.072	0.070	0.080	0.070
	η^2^	0.010	0.003	0.023	<0.001	0.001
	p	0.152	0.473	0.030	0.896	0.610
MD^c^	B	−**0.169**	−0.092	−0.119	−0.037	−0.033
	SE	0.077	0.082	0.078	0.073	0.077
	η^2^	0.023	0.006	0.012	0.001	0.001
	p	0.029	0.266	0.128	0.618	0.667

*FA, fractional anisotropy; MD, mean diffusivity; NAWM, normal appearing white matter; SE, standard error; WMH, white matter hyperintensity*.

*Significant results are indicated in bold, trends are indicated in italic*.

a *Natural log transformed*.

b *Adjusted for age, gender, education, blood pressure, and intracranial volume*.

c *Adjusted for age, gender, education, blood pressure, intracranial volume, NAWM Volume, and WMH Volume*.

## Discussion

We investigated the association between macrostructural and microstructural WM properties of WMH and NAWM per cerebral lobe with core elements of executive function. Next to the replication of well-known findings in the aging literature we found new and important insights into cognitive functioning in healthy older adults. Interestingly, the microstructural characteristics rather than the size of frontal lobe WMH predicted task-switching performance. Conversely, volume but not diffusion characteristics of WMH in the parietal lobe, was associated with task-switching. Thus, WMH severity in the frontal lobe and WMH volume in the parietal lobe are associated with executive functioning in otherwise healthy and highly educated older adults.

### Age effects and regional macro- and microstructural white matter differences

Older age was associated with larger WMH volumes, smaller FA values within NAWM, and larger MD values within NAWM within all cerebral lobes. These findings are in line with previous reports (Barrick et al., 2010; Burzynska et al., 2010). Furthermore, older age was also associated with larger cerebral WMH volumes, which corroborates results from previous studies (Yoshita et al., 2006; Gunning-Dixon et al., 2009; Birdsill et al., 2014). We found the strongest effects of age on NAWM volume and WM microstructure within NAWM in the frontal lobe. In general, age related microstructural WM changes are observed in an anterior-posterior gradient (Pfefferbaum et al., 2005). This means that aging relatively spares the occipital lobe and that most changes occur in the frontal lobe (Salat et al., 2005). Our findings from diffusion values within WMH further support this idea because we found that the lowest FA values in WMH were found in the frontal lobe whereas the lowest MD values were observed in the occipital lobe. In addition, diffusion metrics within WMH were also associated with age, suggesting continued age-related microstructural abnormalities even in areas with frank macrostructural damage. Interestingly, FA values within WMH were higher in older participants. This could be attributed to the breakdown of crossing fibers. Crossing fibers generally have lower FA than fiber bundles that point in the same direction because the directionality of diffusion is less anisotropic. Degeneration of either of these crossing tracts results in increases in FA rather than decreases (Douaud et al., 2011; Teipel et al., 2014). Considering the diffusion values within NAWM we found the lowest FA value within the occipital lobe, which does not fit the anterior-posterior gradient hypothesis. However, FA within NAWM in the occipital lobe was not associated with age, which does support this hypothesis (i.e., the occipital lobe is relatively spared). Potentially, the low FA in occipital NAWM reflects a baseline difference in microstructural properties within NAWM in this cross-sectional study.

The largest relative total WMH volume was found in the frontal lobe followed by the occipital lobe. This is generally in line with former studies, indicating that WMH volume was most extensive in the frontal area (Chen et al., 2006; Yoshita et al., 2006; Meier et al., 2012; Brickman et al., 2014; Tuladhar et al., 2015). After the frontal lobe, the second largest amount of WMH volume is generally found in the parietal lobe (e.g., Chen et al., 2006). Contrasting our lobar WMH volumes with other studies, the larger WMH volume within the occipital lobe compared to parietal regions can possibly be explained by the relatively low WMH burden within the parietal lobe found in our participants. In contrast, WMH volume within the occipital lobe is similar to what has been reported by others. This observation is particularly interesting because larger WMH volume in the parietal lobe has been associated with Alzheimer's disease (Brickman et al., 2014, 2015). Thus, our observation of relatively small parietal lobe WMH volumes in our participants may reflect their brain health and low prevalence of preclinical Alzheimer's disease, which is in line with their high education levels that acts as a preventive measure for neurodegeneration (Orrell and Sahakian, 1995).

Overall, our findings support our hypotheses of gradual deterioration of NAWM and WMH with age and warrant stratified investigation of the association between cognitive functioning and microstructural WM properties of NAWM and WMH.

### Associations between cognition and white matter characteristics

NAWM volume was associated with processing speed and task-switching performance, which is in line with previous reports (e.g., Vernooij et al., 2009). However, we did not see associations between inhibition performance and white matter properties in WMH or in NAWM. However, even though microstructural properties within NAWM have been associated with cognition as well as non-primarily cognitive constructs such as personality disorders and emotion regulation (Kubicki et al., 2005; Spalletta et al., 2013; Ninomiya et al., 2018), we did not find a relationship with our measure of task-switching. This observation stands in contrast to previous studies demonstrating that executive function performance deterioration is associated with microstructural WM changes (Grieve et al., 2007; Madden et al., 2009; Zahr et al., 2009; Parks et al., 2011; Albinet et al., 2012; Soriano-Raya et al., 2014; Tuladhar et al., 2015; Cremers et al., 2016). Similar to our findings, several studies found no associations between inhibitory abilities and WM diffusion properties (O'sullivan et al., 2001; Kennedy and Raz, 2009). The fact that some previous studies did find an association between executive functions and WM microstructure could be because of the non-specific executive functioning measures that were being used. For instance, it could be that some of these measures rely on multiple aspects of executive functioning or even other cognitive capacities (e.g., processing speed). This could thereby falsely suggest that executive functioning in general is associated with WM microstructure. By separating distinct elements of executive functioning (i.e., task-switching and inhibition) we thus show that these specific elements are unrelated to microstructural properties of the NAWM in healthy older adults. Moreover, associations between task-switching and diffusion metrics as reported by Gold et al. (2010) and Perry et al. (2009) might also be attributable to the omission of separation between NAWM and WMH. Perhaps, the association was driven by only one of the types of WM. In contrast to the lack of associations between NAWM diffusion characteristics and executive function performance, processing speed was associated with WM integrity within the NAWM globally but also in the parietal and temporal lobe.

In support of our hypothesis we found that cognitive performance was associated with both volume and microstructural characteristics of WMH. While several studies reported an association between processing speed and/or executive function and WMH volume (Vernooij et al., 2009; Birdsill et al., 2014; Arvanitakis et al., 2016; Luo et al., 2017), several studies have failed to find such an association (for a review see: Mortamais et al., 2013). Interestingly, in our study, only parietal lobe WMH volume was associated with task-switching performance. Regional specificity was also found for diffusion values within WMHs. Whereas task-switching was only associated with diffusion metrics within frontal lobe WMH, processing speed was related to WM microstructure of WMH in the cerebrum and in the parietal lobe. These effects that were small to medium in size (η^2^ = 0.020–0.026) underline that different brain regions are involved in executive functioning and processing speed. This pattern of observations is in line with the prefrontal-executive theory, as well as with a previous study suggesting that processing speed is associated with global WM deterioration, whereas executive function is more often affected by local WM integrity deterioration (Albinet et al., 2012).

Our results emphasize that both severity as well as the amount of white matter deterioration affect cognitive functioning. To date few studies have analyzed diffusion metrics within WMHs, and only Vernooij et al. (2009) compared associations between diffusion properties in WMH to those of NAWM with cognition. They found that diffusion metrics, except for FA within WMH, were associated with processing speed, memory and global cognition after controlling for WMH volume. In contrast, no associations between executive function and microstructural alterations within WMH were shown. However, Vernooij et al. (2009) only looked at global associations and did not stratify their analyses by cerebral lobe. Our findings emphasize the importance of regional analysis, because it demonstrates the relevance of the frontal lobe for executive functioning as well as the impact of the specific contribution of the parietal lobe within cognitive aging.

In general, the disparity between associations of microstructural properties within WMH and NAWM with cognition found in this study emphasizes the importance of separating the two tissue types when studying associations between indices of WM and cognition in the aging brain. This requires the simultaneous collection of T1-weighted data, T2-FLAIR data, and DTI data. However, simply thresholding the diffusion measures using an arbitrary value to discriminate between NAWM and WMH does not seem appropriate since the range of diffusion values within NAWM and WMH overlap. In contrast, combining multimodal imaging enables the automated separation of NAWM and WMH in an appropriate manner. Moreover, we found that distinct elements of executive function are differently associated with WM characteristics. This argues against the use of composite scores for executive function.

### Gender effects

We found that females have significant larger WMH volumes in the cerebrum and in the frontal lobe. Females also have less NAWM volume than males. This finding is in line with previous studies indicating that females have less WM volume (Abe et al., 2010; Rathee et al., 2016) and larger WMH volumes (Sachdev et al., 2009). There were no age^*^gender interaction effects found for either WMH or NAWM volumes, indicating that despite lower NAWM volumes in females, the aging process of WM macrostructural measures does not differ between men and women. This finding is in good correspondence with results from an earlier study indicating that brain atrophy in older age is not modulated by gender (Lemaître et al., 2005). Furthermore, it is important to emphasize that despite the significant explanation of variance by gender, the effect sizes were comparatively small. In our models, which were controlled for ICV, gender explained 1.6–3.3% of variance. When ICV was not included in the model the explained variance of gender increased, explaining up to 26%. Our results are in line with prior findings indicating the importance of ICV correction in volumetric studies when comparing measures between males and females (Jancke et al., 2015).

Within NAWM we found that females have higher MD values than males in the occipital lobe (at baseline) but males seem to have higher rates of MD increase within this region compared to females. Higher rates of MD increase in males than females were also found in the temporal lobe. Previous reports on gender effects and diffusion metrics show that compared to males, females have significantly lower FA values across the brain WM, especially in the temporal lobe (e.g., Hsu et al., 2008; Inano et al., 2011; Rathee et al., 2016). Although we did not find significant gender differences for FA measures, our results depict similar non-significant patterns. Our observed interaction effects of age with WM microstructure indicate that initial microstructural WM differences between males and females are converging during the aging process. However, other studies reporting on gender^*^age interactions found contradictory results. Whereas some studies found no gender^*^age interaction (e.g., Inano et al., 2011) others found that males show significantly increased FA decline (e.g., Kochunov et al., 2012). Although gender differences were reported previously, causes for differences are currently unknown. Interestingly, we also found gender differences and interaction effects for FA within WMH in the parietal lobe. Leading to the conclusion that the gender differences are not restricted to NAWM. In conclusion, our results suggest that gender does not affect the association between WM characteristics and cognition in healthy older age.

### Strengths and limitations

By using a multimodal approach we were able to accurately segment cerebral WM into NAWM and WMH and obtain information about diffusion metrics within these classes. Furthermore, this is the first study that distinguishes between cerebral lobes in the association between WM microstructural parameters within WMH and cognitive performance. A further core strength of the reported study is the highly educated, large and homogenous sample of older participants selected by applying strict eligibility criteria. It is therefore unlikely that our results are substantially confounded by neurodegenerative diseases and thus provide unique insight in to cognitive functioning in healthy aging. In addition, our data was collected using a 3-Tesla scanner, whereas previous studies that differentiated between NAWM and WMHs (Vernooij et al., 2009; Schmidt et al., 2010; Jokinen et al., 2013; Tuladhar et al., 2015), used MRI scanners with lower field strengths. Higher field strengths provide a better signal to noise ratio and thus more power to detect true associations.

The findings of the present study should be interpreted with the following limitations in mind. First, this is a cross-sectional evaluation and therefore no conclusion about causality can be drawn. In addition, executive function scores and processing speed were measured with a single test. Including multiple measures for processing speed or for a distinct element of executive function could yield a more reliable estimate. Executive functions are disproportionally associated with the frontal lobe. Thus, we chose a lobar distinction of WMH. In contrast, several previous studies have separated deep WMH and periventricular WMH. We decided to refrain from this distinction for the following two reasons: First, there is no agreed upon protocol for where the line between periventricular and deep WMH should be drawn. Therefore, such a distinction seems arbitrary to us. Second, 3D reconstructions of WMH indicate that what is often considered “periventricular” and what is considered “deep” is typically part of the same deterioration process but might look distinct because it is often appreciated only on a single slice. Further, we desisted to stratify our analyses by hemisphere because WMH are typically distributed symmetrically over the two hemispheres. Therefore, stratifying does not provide additional information regarding the association of WM properties with cognitive functioning. In addition, the associations between cognition and WM characteristics found in this study did not survive a correction for multiple comparisons. However, we found significant brain-behavioral relations with small to medium effect sizes in a very healthy, highly functioning sample within a narrow age range, after controlling for several covariates. Thus, we are convinced that our observations still provide insight in brain behavioral relationships in healthy aging.

## Conclusion

Executive function and processing speed are selectively associated with microstructural properties of WM in healthy older adults. Both are associated with overall NAWM volume, but only processing speed is related to microstructural properties of NAWM and WMH of the parietal lobe and NAWM microstructural properties of the temporal lobe. Conversely, task-switching but not processing speed is related to microstructural properties of frontal WMHs and WMH volume of the parietal lobe. Our results provide a better understanding of cognitive functioning in healthy older adults because they show that both volume and microstructural properties of WMHs and NAWM are independently associated with cognitive functioning and that these relationships differ per cerebral lobe. Moreover, our findings emphasize the importance of the frontal and parietal lobe in cognition in healthy older adults. Our results show that distinct executive function subcomponents are differently associated with cerebral WM properties. This argues against the use of composite scores that comprise more than one executive function subcomponent.

## Author contributions

SH was responsible for the data acquisition, design of the study, the analysis of the data, the interpretation of data, and wrote the manuscript; VK calculated the LMM and helped with the analysis of the DTI data; SM supervised the data acquisition and also the design of the study, and analysis of the data; CE helped with the data acquisition, and the analysis of data; AN, and AMB processed the FLAIR imaging data, and helped with the analysis of the data; LJ designed the study and supervised the data acquisition and analysis of the data. All authors were involved in the interpretation of the results and drafting and writing the work or revising it critically for important intellectual content. All authors gave their final approval of the version to be published. All authors agree to be accountable for all aspects of the work in ensuring that questions related to the accuracy or integrity of any part of the work are appropriately investigated and resolved.

### Conflict of interest statement

The authors declare that the research was conducted in the absence of any commercial or financial relationships that could be construed as a potential conflict of interest.
